# Impact of serum cholesterol esterification rates on the development of diabetes mellitus in a general population

**DOI:** 10.1186/s12944-018-0822-5

**Published:** 2018-07-28

**Authors:** Shin-ichiro Tanaka, Yoshio Fujioka, Takeshi Tsujino, Tatsuro Ishida, Ken-ichi Hirata

**Affiliations:** 1 The Department of Internal Medicine, Toyooka Hospital Hidaka Medical Center, Toyooka, Hyogo Japan; 2 Division of Clinical Nutrition, Faculty of Nutrition, Kobe Gakuin University, Kobe, Japan; 3 Department of Pharmacy, School of Pharmacy, Hyogo University of Health Sciences, Kobe, Japan; 4 The Division of Cardiovascular Medicine, Kobe University Graduate School of Medicine, Kobe, Japan

**Keywords:** Lecithin:cholesterol acyltransferase, Diabetes, Cholesterol metabolism, Epidemiology, Insulin resistance, Lipotoxicity, Cohort study

## Abstract

**Background:**

Lecithin:cholesterol acyltransferase (LCAT) plays an important role in cholesterol esterification in serum. Serum LCAT activity is elevated in patients with serum high triglyceride and low high-density lipoprotein-cholesterol (HDL-C) concentrations, both of which are related to metabolic syndrome and subsequent diabetes mellitus, referred to as lipotoxicity. We hypothesized that increased serum LCAT activity could predict future risk of diabetes mellitus in a general Japanese population.

**Methods:**

We prospectively studied 1496 individuals aged 20–86 years without histories of diabetes mellitus at baseline. Serum lipid concentrations, glucose parameters, and LCAT activity measured as the serum cholesterol esterification rate, were evaluated.

**Results:**

During 11 years of follow-up, 46 newly diagnosed patients with diabetes mellitus were reported. After adjustment for plasma glycosylated hemoglobin A1c (HbA1c) levels, the relative risks (RRs) for the development of diabetes mellitus were 5.45 [95% confidence interval (95% CI) 2.37–12.55; *P* <  0.001] for body-mass index, 0.22 (95% CI, 0.09–0.53; *P* = 0.001) for HDL-C, 4.81 (95% CI, 1.96–11.77; *P* = 0.001) for triglyceride, and 4.64 (95% CI, 1.89–11.41; *P* = 0.001) for LCAT activity. After adjustment for HbA1c, total cholesterol, triglyceride, HDL-C, phospholipid, and free fatty acid levels, the RR of LCAT activity for future risk of diabetes mellitus remained significant (RR, 4.93; 95% CI,1.32–18.41; *P* = 0.018). In this analysis, we found a significant association between LCAT activity and risk of diabetes mellitus in men but not in women.

**Conclusion:**

Increased serum cholesterol esterification rate is a potent predictor for future diabetes mellitus.

## Background

Dyslipidemic conditions, represented by serum high TG, low high-density lipoprotein-cholesterol (HDL-C), and increased free fatty acid concentrations, are associated with insulin resistance and subsequent diabetes mellitus [1–4]. The accumulation of lipids and lipid metabolites, such as fatty acyl-CoA, diacylglycerol, and ceramide, in circulating blood and non-adipose tissues deteriorates insulin signaling and promotes beta-cell apoptosis, referred to as lipotoxicity [5–8]. Nevertheless, the detailed mechanisms by which lipids cause diabetes mellitus in general populations have not yet been fully elucidated. Lecithin: cholesterol acyltransferase (LCAT) is an enzyme that catalyzes the sn-2 position of phosphatidylcholine and the 3-beta-hydroxyl group of cholesterol to form cholesterol ester and lysophosphatidylcholine [9]. This reaction occurs in high-density lipoproteins (HDL) and is therefore thought to be important in HDL maturation and subsequent reverse cholesterol transport to the liver [9, 10]. However, we have previously reported that increased serum LCAT activity measured as serum cholesterol esterification rates were associated with future risk of coronary heart disease and sudden death in a general population [11]. In that study, we also observed that serum LCAT activity was highly correlated with increased waist circumference, increased serum TG concentration, and decreased serum HDL-C concentration, all of which are components of metabolic syndrome and are related to insulin resistance [12]. A number of previous studies also reported similar results [13–16]. These findings may indicate that increased LCAT activity is closely related to increased risk factors for type 2 diabetes mellitus as well as coronary heart disease, suggesting that both diseases have common antecedents [2, 7, 12]. Furthermore, Recently, Ng et al. have reported a significant decrease in fasting insulin and glucose levels in LCAT- and low-density lipoprotein receptor-knockout mice, despite high serum TG concentration [17, 18]. This result suggests that LCAT may be closely related to insulin resistance and subsequent diabetes mellitus. Taken together, we hypothesized that increased serum LCAT activity is predictive of future risk of diabetes mellitus. Here, we examined the relationship between baseline LCAT activity, measured as serum cholesterol esterification rate, and future risk of diabetes mellitus in a general Japanese population.

## Methods

### Study population

The Hidaka Cohort Study is a population-based cohort study in the town of Hidaka, a typical Japanese rural community [11, 19]. The baseline survey was conducted in 1993. There were 2155 total participants aged 20–93 years at baseline.

### Follow-up and outcomes

**Fig. 1:**
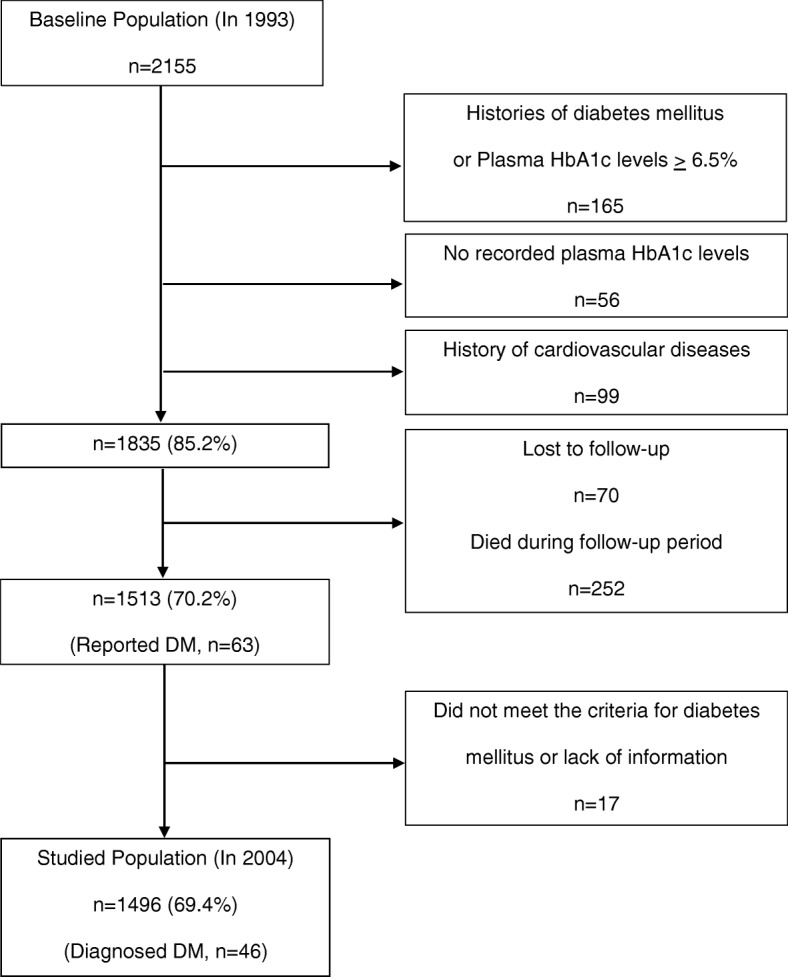
Selection procedure

We initiated a follow-up study in 2004. We used mailed questionnaires or telephone interviews to measure the incidence of death, stroke, myocardial infarction, cancer, and newly developed diabetes mellitus during the follow-up period. Among them, 165 participants had histories of diabetes mellitus or whose plasma glycosylated hemoglobin A1c (HbA1c) levels were ≥ 6.5% at baseline. In addition, 56 participants did not have recorded plasma HbA1c levels, and 99 participants had histories of cardiovascular diseases. Of the participants, 70 were lost to follow-up, and 252 died during the follow-up period; these participants were excluded from the analysis. Among the remaining 1513 participants, 63 participants reported newly developed diabetes mellitus during the follow-up period. For these 63 participants, we further confirmed the diagnosis of diabetes mellitus using medical records. The criteria were as follows; plasma HbA1c level ≥ 6.5%, or fasting plasma glucose concentration ≥ 7.0 mmol/L, or 2-h plasma glucose concentration during a 75 g oral glucose tolerance test ≥ 11.1 mmol/L, or casual blood glucose concentration ≥ 11.1 mmol/L, or current or past use of antidiabetic agents including insulin, sulfonylureas, biguanides, alpha-glucosidase inhibitors, meglitinides, dipeptidyl peptidase-4 inhibitors, and thiazolidinediones. Eleven subjects did not meet the criteria and 6 subjects were not determined as having diabetes mellitus due to lack of information. Therefore, these 17 subjects were excluded from the analysis. The remaining 1496 participants consisting of 929 women and 567 men aged 55.7 ± 14.1 years (mean ± SD, range 20–86 years) were eligible for the study. (See Fig. 1).

### Laboratory procedures

Most of the blood samples were drawn within eight hours after the participants’ last meal; thus, the samples were mainly obtained in a non-fasted state. Serum LCAT activity was determined as described previously [11]. We used a self-substrate method, where decreases in free cholesterol concentrations were measured enzymatically after incubating sera with synthetic dipalmitoyl lecithin using a commercially available kit (Nescoat LCAT kit-S, Alfresa Pharma, Osaka, Japan) based on the method by Nagasaki and Akanuma [20]. The present method is simple and rapid as compared with the traditional endogenous substrate methods using gas-liquid chromatography. Therefore it is suitable for assaying a large number of samples from the cohort and is easy to employ in clinical laboratories. In brief, 0.5 ml of serum was added to 0.3 ml of 2.5 mg/ml lecithin solution and mixed gently. 0.2 ml of the mixture was transferred into a test tube and stored in a refrigerator as a control (A). The remaining mixture was incubated at 37 °C for 2 h. 0.2 ml of the mixture was transferred into another test tube (B). 3.0 ml of color reagent for cholesterol measurement was added to both (A) and (B), and incubated at 37 °C for 20 min. The difference in absorbance between (A) and (B) at 600 nm was determined, and the decrease of cholesterol concentration was calculated as an LCAT activity (n moles/ml/hr). 1000 n mole/ml of purified cholesterol was used as a reference for calculating cholesterol esterification rate. Therefore, the present assay is a modification of the endogenous substrate assay. To test whether adding dipalmitoyl lecithin into the assay system affects the result of the analysis, we examined the relationship between the present method and the radio-labeled endogenous substrate method by Stokke and Norum [21]. We confirmed a high correlation between the present method and the traditional endogenous substrate method (*R* = 0.69, *P* = 0.0087, *n* = 13, unpublished data). Furthermore, The LCAT activities measured by the present method are highly correlated with those measured by the endogenous substrate method using gas-liquid chromatography (*R* = 0.829, *P* <  0.001, *n* = 22) [20], with those measured by the exogenous substrate method (*R* = 0.99, *P* <  0.001, *n* = 38) [22], and with the LCAT mass concentrations measured by the enzyme-linked immunosorbent assay (*R* = 0.864, *P* <  0.001, *n* = 45) [23]. Therefore, this method is well-validated and reliable for this study. Serum total cholesterol (TC) and TG concentrations were determined by enzymatic methods. High-density lipoprotein-cholesterol (HDL-C) concentrations were determined using the phosphotungstic acid magnesium chloride precipitation method. Plasma HbA1c levels were measured using high-performance liquid chromatography. Serum immunoreactive insulin (IRI) levels were measured using an enzyme-linked immunosorbent assay. We calculated the serum non-HDL-C concentration as the difference between serum TC and HDL-C concentrations.

### Blood pressure measurement and lifestyle factor assessment

Community nurses interviewed the participants for information on current and past health conditions, medications, and lifestyle. Blood pressure was measured with a standard mercury sphygmomanometer on the right arm after the participants had been sitting at rest for at least 3 min. BMI was calculated as weight in kilograms divided by the square of height in meters.

### Statistical analyses

We first calculated the means ± standard deviations, medians, and proportions of potential diabetic risk factors at baseline for the 1496 participants. Student’s t-test, the Mann-Whitney nonparametric test, and the chi-square test were used to examine differences between two groups. We divided the lipid parameter distributions into quartiles, and the quartile-specific relative risks (RRs) and confidence intervals (CIs) for future risk of diabetes mellitus were estimated by multiple logistic regression adjusted for HbA1c levels, lipid concentrations, and their parameters. All of the *P* values were two-sided, and P values less than 0.05 were considered statistically significant. All of the analyses were performed with SPSS 11.01 J software for Windows (SPSS, Japan, Tokyo, Japan).

## Results

### Baseline characteristics of the study cohort

Table 1 shows baseline characteristics of the study cohort. Blood pressure, TG concentration, and LCAT activity were higher in men than in women. TC, HDL-C, and Immunoreactive insulin (IRI) levels were higher in women than in men. We found no significant differences in BMI and blood glucose levels between men and women.

**Table 1:** Baseline characteristics of the study cohort

Characteristics	Total	Women	Men	*P* Value*
Number of subjects	1496	929	567	
Age, years	55.7 ± 14.1	55.9 ± 14.1	55.3 ± 13.9	0.40
History of hypertension, %	30.3	31.8	28.0	0.12
Current smoker, %	20.6	1.9	51.1	< 0.001
BMI, kg/m^2^	22.5 ±2.9	22.5 ± 3.0	22.5 ± 2.8	0.85
Waist circumference, cm	72.2 ± 8.1	69.6 ± 7.1	76.8 ± 7.7	< 0.001
Waist/Hip	0.81± 0.07	0.78 ±0.06	0.85 ± 0.06	< 0.001
SBP, mmHg	132 ± 21	131 ± 21	134 ± 20	0.002
DBP, mmHg	77 ± 12	76 ± 12	79 ± 12	< 0.001
HbA1c, %	5.1 ± 0.4	5.1 ± 0.4	5.2 ± 0.4	< 0.001
Glucose, mmol/L	5.52 ± 0.70	5.52 ± 0.68	5.52 ± 0.74	0.93
IRI, U/mL	2.9 (1.7–5.5)	3.3 (1.9–5.6)	2.5 (1.3–4.8)	< 0.001
TC, mmol/L	5.16 ± 0.93	5.31 ± 0.93	4.92 ± 0.87	< 0.001
HDL-C, mmol/L	1.54 ± 0.38	1.58 ± 0.37	1.47 ± 0.37	< 0.001
Non-HDL-C, mmol/L	3.62 ± 0.38	3.73 ± 0.99	3.45 ± 0.94	< 0.001
TG, mmol/L	0.99 (0.71–1.42)	0.95 (0.68–1.33)	1.11 (0.78–1.61)	< 0.001
Phospholipids, mg/dL	220 ± 33	223 ± 32	214 ± 33	< 0.001
Free fatty acids, Eq/L	561 (265–854)	629 (292–912)	486 (231–755)	< 0.001
LCAT activity, nmol mL^-1^ hr^-1^	76.9 ± 21.4	75.6 ± 21.2	79.0 ± 21.5	0.003

Values are expressed as means ± standard deviations except IRI, TG, and free fatty acids which are given as the medians and the interquartile ranges in parentheses

^*^P < 0.05 is considered to indicate statistically significant differences between men and women. The Mann-Whitney nonparametric test was used for the differences of IRI, *TG* and free fatty acids. The other continuous variables were estimated by Student’s t-test

*BMI* body mass index, *SBP* systolic blood pressure, *DBP* diastolic blood pressure, *HbA1c* glycosylated hemoglobin A1c, *IRI* immunoreactive insulin, *TC* total cholesterol, *HDL-C* high-density lipoprotein-cholesterol, *TG* triglyceride, *LCAT* lecithin cholesterol acyltransferase

During the 11.0 years of follow-up, 46 diabetes mellitus cases (21 women, 25 men) were reported from the 1496 participants.

### Baseline characteristics of newly diagnosed diabetes and non-diabetes subjects from the total cohort

Table 2 shows baseline characteristics of newly diagnosed diabetes and non-diabetes subjects from the total cohort. BMI, waist circumference, SBP, HbA1c, glucose, and IRI levels were significantly higher in newly diagnosed diabetes subjects than in non-diabetes subjects. Among lipid markers, non-HDL-C, TG concentrations, and LCAT activity were significantly higher in newly diagnosed diabetes subjects, whereas HDL-C was significantly lower in newly diagnosed diabetes subjects.

**Table 2:** Baseline characteristics of newly diagnosed diabetes and non-diabetes subjects from the total cohort

Characteristics	Non-diabetes	Diabetes	*P* Value ^*^
Number of subjects	1450	46	
Age, years	55.6 ± 14.1	56.8 ± 10.9	0.599
Male sex (%)	37.4	54.3	0.02
History of hypertension, %	30.1	39.1	0.19
Current smoker, %	20.3	30.4	0.09
BMI, kg/m^2^	22.4 ± 2.8	25.3 ± 3.0	< 0.001
Waist circumference, cm	72.0 ± 7.9	79.8 ± 10.2	< 0.001
Waist/Hip	0.81 ± 0.06	0.86 ± 0.08	< 0.001
SBP, mmHg	132 ± 21	141 ± 20	0.003
DBP, mmHg	77 ± 12	81 ± 13	0.01
HbA1c, %	5.1 ± 0.4	5.6 ± 0.4	< 0.001
Glucose, mmol/L	5.51 ± 0.68	6.03 ± 1.07	< 0.001
IRI, U/mL	2.9 (1.6–5.4)	4.5 (2.9–8.7)	0.002
TC, mmol/L	5.15 ± 0.91	5.47 ± 1.23	0.02
HDL-C, mmol/L	1.54 ± 0.38	1.31 ± 0.32	< 0.001
Non-HDL-C, mmol/L	3.61 ± 0.97	4.16 ± 1.28	< 0.001
TG, mmol/L	0.98 (0.71–1.40)	1.61 (1.04–2.13)	< 0.001
Phospholipids, mg/dL	219 ± 32	232 ± 45	0.01
Free fatty acids, Eq/L	563 (265–852)	498 (246–942)	0.95
LCAT activity, nmol mL^−1^ h^− 1^	76.4 ± 21.2	92.0 ± 21.2	< 0.001

Values are expressed as means ± standard deviations except IRI, TG, and free fatty acids which are given as the medians and the interquartile ranges in parentheses

^*^ *P* < 0.05 is considered to indicate statistically significant differences between men and women. The Mann-Whitney nonparametric test was used for the differences of IRI, *TG* and free fatty acids. The other continuous variables were estimated by Student’s *t*-test

*BMI* body mass index, *SBP* systolic blood pressure, *DBP* diastolic blood pressure, *HbA1c* glycosylated hemoglobin A1c, *IRI* immunoreactive insulin, *TC* total cholesterol, *HDL-C* high-density lipoprotein-cholesterol, *TG* triglyceride, *LCAT* lecithin cholesterol acyltransferase

### Univariate and HbA1c-adjusted analysis of risk factors and incidence of diabetes mellitus

Table 3 lists the quartile-specific relative risks (RRs) for the future risk of diabetes mellitus. Using univariate analysis, we found that the significant risk factors for diabetes mellitus according to the highest versus the lowest quartiles included BMI, waist circumference, waist to hip ratio, systolic blood pressure, diastolic blood pressure, glycosylated hemoglobin A1c (HbA1c), glucose, IRI, HDL-C, TG, non-HDL-C, and LCAT activity. Therefore, most lipid markers were positively associated with the risk of diabetes mellitus except for HDL-C, which was inversely associated with the risk. Among various metabolic factors, increased body weight, abdominal obesity, increased TG, and decreased HDL-C were more likely to be associated with the risk of diabetes mellitus, whereas systolic and diastolic blood pressures were less likely to be associated with the risk. In this analysis, HbA1c as a glucose metabolism parameter was most associated with the risk of diabetes mellitus. Therefore, we included HbA1c as a covariate to the analysis to exclude the potential effects of hyperglycemia on the development of diabetes mellitus. After adjustment for HbA1c levels, we found significant risk factors for diabetes mellitus including BMI, waist circumference, waist to hip ratio, systolic blood pressure, IRI, HDL-C, TG, and LCAT activity. Among these, BMI, increased TG, decreased HDL-C, and increased LCAT activity were associated with the risk of diabetes mellitus with relatively higher RRs than increased blood pressure in this population.

**Table 3:** Univariate and HbA1c-adjusted analysis of risk factors and incidence of diabetes mellitus

Variables	Univariate		HbA1c adjusted	
	RR^*^ (95%CI)	*P* value	RR^*^ (95%CI)	*P* value
Age	1.01 (0.99–1.03)	0.60	0.98 (0.96–1.00)	0.09
Man	1.99 (1.11–3.60)	0.02	1.60 (0.87–2.93)	0.13
History of Hypertension	1.50 (0.82–2.73)	0.19	1.39 (0.75–2.58)	0.33
Current smoker	1.72 (0.91–3.27)	0.10	1.39 (0.72–2.71)	0.33
BMI	8.04 (2.81–23.07)	<0.001	7.52 (2.58–21.90)	<0.001
Waist circumference	5.57 (2.12–14.63)	<0.001	4.23 (1.58–11.30)	0.004
Waist/hip	6.10 (2.09–17.80)	0.001	3.76 (1.26–11.22)	0.02
SBP	3.99 (1.47–10.81)	0.006	3.06 (1.11–8.45)	0.03
DBP	2.26 (1.06–4.82)	0.04	2.09 (0.96–4.57)	0.06
HbA1c	16.70 (3.98–70.14)	<0.001		
Glucose	3.34 (1.32–8.47)	0.01	2.11 (0.81–5.50)	0.13
IRI	2.19 (0.93–5.13)	0.07	2.58 (1.07–6.23)	0.04
TC	1.75 (0.79–3.88)	0.17	1.17 (0.52–2.67)	0.70
HDL-C	0.16 (0.05–0.46)	0.001	0.18 (0.06–0.54)	0.002
TG	10.50 (3.17–34.76)	<0.001	8.11 (2.42–27.22)	0.001
Non-HDL-C	3.11 (1.31–7.41)	0.01	2.11 (0.86–5.14)	0.10
Phospholipids	3.30 (1.20–9.10)	0.02	2.66 (0.95–7.45)	0.06
Free fatty acids	1.00 (0.47–2.13)	1.00	1.01 (0.46–2.20)	0.98
LCAT activity	9.42 (2.82–31.39)	<0.001	7.37 (2.18-24.89)	0.001

^*^RR indicates a relative risk for diabetes mellitus in quartile 4 subjects as compared with quartile 1 subjects for continuous variables except for age, which is expressed as a risk per one year increment. RR of Man indicates the risk of diabetes mellitus of men compared with that of women

*BMI* body mass index, *SBP* systolic blood pressure, *DBP* diastolic blood pressure, *HbA1c* glycosylated hemoglobin A1c, *IRI* immunoreactive insulin, *TC* total cholesterol, *HDL-C* high-density lipoprotein-cholesterol, *TG* triglyceride, *LCAT* lecithin cholesterol acyltransferase

### Lipid- and HbA1c-adjusted multivariable analysis of the risk of diabetes mellitus according to baseline LCAT activity

To determine whether the predictive value of LCAT activity was independent of the other lipid markers, we conducted multivariable analysis adjusted for these lipid markers in addition to HbA1c levels (Table 4). After adjusting for HbA1c, TC, TG, HDL-C, phospholipids, and free fatty acids, the RR of LCAT activity was 4.93 (95% CI, 1.32–18.41; *P* = 0.018). Therefore, increased LCAT activity was significantly associated with the risk of diabetes mellitus independent of other lipid markers. However, in the analysis performed on men and women separately, we found a significant association between baseline LCAT activity and the future risk of diabetes mellitus only in men (RR 9.15; 95% CI, 1.02–81.95; *P* = 0.048 for men and RR, 2.67; 95% CI, 0.47–15.41; *P* = 0.27 for women). When we performed a multivariable analysis including BMI as a covariate in addition to lipids and HbA1c levels, we could not find any significant associations between LCAT activity and risk of diabetes mellitus in this analysis (data not shown).

**Table 4:** Lipid- and HbA1c-adjusted multivariable analysis of the risk of diabetes mellitus according to baseline LCAT activity

	Number of subjects	RR^*^	95%CI	*P* Value
Women	929	2.67	0.47–15.41	0.27
Men	567	9.15	1.02–81.95	0.048
Total	1496	4.93	1.32–18.41	0.018

^*^RR indicates a relative risk for diabetes mellitus in quartile 4 subjects as compared with quartile 1 subjects of LCAT activities, adjusted for HbA1c, TC, TG, HDL-C, phospholipids, and free fatty acids

*LCAT* lecithin cholesterol acyltransferase, *HbA1c* glycosylated hemoglobin A1, *TC* total cholesterol, *TG* triglyceride, *HDL-C* high-density lipoprotein cholesterol

## Discussion

In this population-based cohort study, most of the variables predicting future diabetes mellitus are also known to be risk factors for coronary heart disease. Among these, serum LCAT activities were significantly associated with future risk of diabetes mellitus. This association was independent of other lipid markers, including TC, HDL-C, TG, phospholipids, and free fatty acids. Therefore, increased serum LCAT activities may play a pivotal role in the development of both diabetes mellitus and coronary heart disease among various lipid markers [11]. A number of studies have revealed that increased plasma TG concentrations are associated with higher plasma LCAT activity [11, 13–16]. However, in LCAT-deficient humans and animals, plasma TG concentrations were shown to be increased [17, 24–27]. Among these studies, Ng et al. reported significant decreases in fasting plasma insulin and glucose concentrations and an improvement of insulin signaling pathways in LCAT- and LDL receptor-double-knockout mice, despite increased plasma TG concentrations [17, 18]. Therefore, it is unlikely that increased serum LCAT activity causes hypertriglyceridemia [17, 24]; instead, hypertriglyceridemia may facilitate LCAT activity [16]. Li et al. have reported a mechanistic explanation for these results, that the impaired expression of hepatic insulin receptor substrate 2 and its downstream transcription factors promote insulin resistance, at least partially through LCAT-dependent pathways [28]. Taken together, hypertriglyceridemia may cause insulin resistance through LCAT dependent pathways. In addition, lysophosphatidylcholine, which is generated by LCAT, has also been shown to impair insulin signaling [29, 30]. These deleterious effects of lysophosphatidylcholine may contribute to the development of diabetes mellitus.

Increased plasma free fatty acid levels are a cause of insulin resistance [4–6]. However, we did not identify any association between serum free fatty acid levels and the risk of diabetes mellitus. In our study, most of the blood samples were drawn within 8 h of the participants’ last meal. Therefore, dietary fatty acids might influence the association between serum free fatty acid concentrations and the risk of diabetes mellitus. Furthermore, recent studies have also demonstrated that saturated fatty acids can compromise insulin signaling, whereas polyunsaturated fatty acids diminish the deleterious effects of saturated fatty acids on insulin signaling [31, 32]. Therefore, not only fatty acid concentrations but also compositions may be critical for insulin resistance.

We found a significant increased risk of diabetes mellitus in the male population who had higher serum cholesterol esterification rates but not in the female population. These results are different from the results in the animal study based on LCAT- and low-density lipoprotein receptor-double-knockout mice, which indicated improved insulin sensitivities only in female mice [33]. However, we should consider that measuring serum cholesterol esterification rates in a human population is different from a genetically-modified LCAT mass expression in a specific animal model. Therefore, comparing the results from the different subjects and methods may not be appropriate. Nevertheless, we also showed that increased cholesterol esterification rates were associated with future coronary heart disease and sudden death in a general population, which was especially evident in a female population. These gender-specific phenomena have not yet been fully explained. Increased serum cholesterol esterification rates and risks of coronary heart disease and diabetes mellitus are substantially related. However, the mechanisms seem to be different. Further studies are needed to clarify these gender-specific phenomena.

Our study has limitations. The incidence rate of diabetes mellitus in our study seems to be smaller than that of previous reports [34, 35]. However, the cases of diabetes mellitus in our study were identified by self-reported questionnaires. The recent meta-analysis study has shown that the incidence rates of diabetes based on self-reports are smaller than those of laboratory data-based diabetes [36]. In addition, the initial cases of self-reported diagnosed diabetes mellitus in our study were 63 out of 1513 individuals during 11 years of follow-up. Therefore, the incidence rate is not notably different from those of the Japanese population [37–39] or those of the US population based on self-reported diagnosed diabetes [40]. In addition, we further confirmed the diagnosis of diabetes mellitus for each subject using medical records and excluded 17 cases due to not meeting the criteria or due to lack of laboratory tests. This procedure may also contribute to the smaller incidence rate of diabetes mellitus and result in a statistically underpowered analysis.

## Conclusions

Increased serum LCAT activities, measured as the serum cholesterol esterification rates, are associated with the future risk of diabetes mellitus in the general population. This association is significant even after adjustment for HbA1c levels and lipid parameters. Our study indicates that among various lipid markers, increased serum LCAT activity may play a pivotal role in the development of diabetes mellitus in the general population.

## Abbreviations

BMI: Body-mass index
DBP: Diastolic blood pressure
HDL-C: High-density lipoprotein-cholesterol
HbA1c: Glycosylated hemoglobin A1c
IRI: Immunoreactive insulin
RR: Relative risk
SBP: Systolic blood pressure
TC: Total cholesterol
95%CI: 95% Confidence interval
